# Enzymatic characterization and polyurethane biodegradation assay of two novel esterases isolated from a polluted river

**DOI:** 10.1371/journal.pone.0327637

**Published:** 2025-07-23

**Authors:** Arianna Soto-Hernández, Luis Felipe Muriel-Millán, Adolfo Gracia, Alejandro Sánchez-Flores, Liliana Pardo-López

**Affiliations:** 1 Departamento de Microbiología Molecular, Instituto de Biotecnología, Universidad Nacional Autónoma de México, Cuernavaca, Morelos, México; 2 Instituto de Ciencias del Mar y Limnología, Universidad Nacional Autónoma de México, Coyoacán, México; 3 Unidad Universitaria de Secuenciación Masiva y Bioinformática, Universidad Nacional Autónoma de México, Cuernavaca, Morelos, México; Universidade Estadual de Ponta Grossa, BRAZIL

## Abstract

The environmental ubiquity of plastic materials generates global concern, pollution, and health problems. Microorganisms and enzymes with plastic biodegradation potential are considered as environmentally friendly alternatives to address these issues. Interestingly, polluted environments exert selective pressure on native microbial communities that have the metabolic capacity to tolerate and transform different contaminants, including plastics. A number of enzymes have been described as polyurethane degraders. However, some of them do not possess complete characterization or efficient degradation rates. Hence, there is still a need to identify and characterize efficient enzymes for application in green processes for plastic recycling. Here, we used an environmental DNA sample isolated from the sediments of a polluted river in Mexico (Apatlaco River), which was used to construct a metagenomic fosmid library to explore the metabolic potential of microbial communities for polyurethane biodegradation. Functional screenings were performed on agar media containing the polyester polyurethane Impranil DLN (Impranil), and positively selected fosmid DNA was identified and sequenced by Illumina. Bioinformatic analyses identified two *Acinetobacter* genes (*epux1* and *epux2)* encoding alpha/beta hydrolases. The genes were heterologously expressed to determine the capacity of their encoded proteins for Impranil clearing. Both Epux1 and Epux2 enzymes exhibited Impranil cleavage at 30 °C and 15 °C and ester group modifications were validated by infrared spectroscopy. Furthermore, the release of building blocks of the polymer was determined by GC-MS analysis, thus indicating their esterase/polyurethanase activity. Overall, our results demonstrate the potential of these novel bacterial enzymes for the hydrolysis of polyurethane with potential applications in the circular plastics economy.

## Introduction

Polyurethanes (PU) are a family of polymers with a wide range of structural properties and potential for multiple applications (furniture, biomedical devices, paints and coatings). This functional versatility places PU plastics as the 6th most produced plastic in the world [1]. However, PU can’t be easily recycled or reused due to their physicochemical changes when heated. As a result, its residues are mainly incinerated or chemically recycled. These methods are ecologically unsustainable since high temperatures and energy inputs are required [2,3]. Enzymatic treatment has emerged as a promising green method for recycling plastics, including PU, because of the mild reaction conditions which could be advantageous compared to chemical methods. Currently, the main known enzymatic activities related to PU biodegradation are lipase/esterase, amidase, and protease.

Despite several reports of bacteria capable of growing in PU as a sole carbon source such as some species of *Pseudomonas*, *Bacillus, Alicycliphilus*, *Halopseudomonas*, among others, it is difficult to identify the specific enzymes responsible for breaking down PU [4,5], interfering with the deciphering of the metabolic pathways involved in plastic metabolism. It has been reported and proposed that extracellular esterases act mainly during the first stage of biodegradation releasing oligomers from the polymer that can be further metabolized by other enzymes such as amidases [6–8]. However, due to the diversity and complexity of PUs, further research is necessary to identify efficient enzymes that could be applied to the biodegradation of specific types of PU. Currently, the majority of research in this field is conducted on a laboratory scale, focused on enzymes capable of depolymerizing and generating products with lower molecular weights. Still, in some cases, this occurs unspecifically [9] or at low rates. On the other hand, little is known about the enzymatic polymer hydrolysis under environmental fluctuating conditions such as pH and temperature.Therefore, versatile enzymes that work in a wide range of pH and temperature would be good candidates for the industrial processing of PU residues.

Environmental pollution can act as a selective pressure for microorganisms promoting the degradation of xenobiotics. This has been reported for different contaminants such as chlorinated aromatics [10], herbicides [11], hydrocarbons [12], and plastics [13–15]. As an example, the Apatlaco River located in the state of Morelos, Mexico, flows through ten municipalities and is of great ecological and socioeconomic importance. Nevertheless, it is considered a river with critical levels of contamination [16]. It receives 321 official wastewater discharges from industrial, domestic and agricultural sources, resulting in the presence of a large quantity and diversity of contaminants [17]. Previous studies have demonstrated the interesting metabolic potential of the bacterial communities of the Apatlaco River, which might be due to the constant interaction with xenobiotics [18], but to our knowledge, no experimental studies have been performed to test this potential.

The present work aimed to explore and evaluate the metabolic potential of bacterial communities from the Apatlaco River, specifically for PU biodegradation. We used a functional metagenomic approach based on fosmid libraries from environmental DNA (eDNA), followed by sequencing and bioinformatic analyses to identify and select for cloning genes encoding enzymes with the capacity for PU biodegradation. We report two novel esterases capable of hydrolyzing the polyester PU Impranil-DLN. The purified recombinant enzymes hydrolyzed *p*-nitrophenyl esters from C4 to C12 and exhibited a high activity on Impranil at temperatures of 15 and 30°C, and at pH values of 7 and 9. The versatility of these enzymes makes them promising candidates for industrial applications.

Our approach allowed us to efficiently identify new enzymes from eDNA with PU-degrading activity, which contributes to expanding the knowledge on the mechanisms involved in PU biodegradation, and to the design of efficient strategies for biochemical recycling.

## Materials and methods

### Strains, growth, and culture conditions

*Escherichia coli* EPI300-T1^R^ (Epicentre ^TM^, Biosearch Technologies ^TM^) was used for metagenomic library construction and *E. coli* BL21 (DE3) was used for recombinant protein expression. Both strains were grown in LB medium under aerobic conditions at 37 °C.

### Isolation of eDNA

Sediment samples were collected from the Apatlaco River, in Xochitepec, Morelos, Mexico (18°47’02.7“N 99°14’12.7”W), no permits were required. The eDNA was isolated using the Dneasy® PowerMax® Soil Kit (Qiagen) following the manufacturer’s protocol with some modifications. Approximately 5 g of sediment samples were loaded into 50-ml tubes and then incubated in a water bath at 65 °C, with manually shaking every 5 minutes for 30 minutes. The purpose of this modification was to preserve the DNA integrity. Additionally, in the elution step, the buffer was heated at 60 °C, added to the column, and then incubated for ten minutes before centrifugation, which aimed to obtain a higher DNA yield. After elution, the eDNA was concentrated to 1 mL in a centrifugal vacuum concentrator at 45 °C. Finally, the DNA integrity was assessed by electrophoresis in 0.5% agarose gel, at 70 V and 2 h of runtime.

### Metagenomic library construction

The metagenomic library was constructed using the CopyControl HTP Fosmid Library Production Kit with pCC2FOS vector (Biosearch Technologies ^TM^), according to the manufacturer’s instructions. Briefly, eDNA was mechanically sheared by passing it through a 200 μL pipette tip, aspirating and expelling the DNA approximately 50 times. A repair step was subsequently carried out to obtain blunt-ended, 5′-phosphorylated DNA. After low-melting agarose gel electrophoresis (60 V, 6 h), 30–40 kb DNA fragments were selected using a fosmid with a 40 kb insert as a control. The fragments were gel purified for ligation into the fosmid vector pCC2FOS.

Fosmid ligations were packaged into phage particles for the transduction in *E. coli* EPI300-T1^R^. Transformant clones were selected on plates containing LB agar with 12.5 µg mL^-1^ chloramphenicol (Cm), picked, and resuspended in LB with 12.5 µg mL^-1^ Cm and 20% glycerol. Aliquots at a density of ~200 colony-forming units (CFU) were transferred to 96-well plates to organize and store the library into pools.

### Functional parallel screening for esterase and PU degrading activity

Aliquots of 5 µL from each pool were used for tenfold serial dilutions and 600 uL of dilutions 10^−7^ were plated on LB agar with 12.5 µg mL^-1^ Cm. The plates were incubated overnight to obtain individual clones. Each clone was then streaked on LB agar with 12.5 µg mL^-1^ Cm, 0.01% arabinose and 0.5 mM IPTG, containing either 0.3% PU water dispersion Impranil DLN ® (Covestro AG) or 1% tributyrin. A clone containing a fosmid control was also streaked in each plate as a negative control to validate the screenings. The plates were incubated at 37 °C for 3 days, followed by incubation at 4 °C until clearing halos formation.

### DNA sequencing and bioinformatic analysis

Fosmid DNA from clone *1–19* selected from the parallel screening was sequenced using the Illumina Nextera XT DNA Library Preparation Kit and the NextSeq500 platform using a 150-cycle kit with a pair-end configuration. DNA sequences were assembled using the SPAdes algorithm with the following parameters: -k 21,31,41,51,61,71 --careful -t 8 [19]. Gene annotation was carried out with the Bacterial and Viral Bioinformatics Resource Center (BV-BRC) online server [20]. We focused the search on genes encoding hydrolytic enzymes that could attack polyester PU bonds, like alpha/beta hydrolases. An alignment of selected genes and 22 other sequences of characterized lipolytic enzymes was performed with the software MEGA 11 [21] using the Muscle aligner [22] to build a maximum likelihood phylogenetic tree using IQtree [23] with automatic selection for the substitution model and 1000 bootstrap replicates.

Tridimensional protein models were generated with AlphaFold 3 [24]. The predicted models were subjected to a round of energy minimization with the YASARA server [25] and evaluated with the PROCHECK and ERRAT test programs (https://saves.mbi.ucla.edu/).

### Gene cloning and protein expression

The *epux*1 and *epux*2 genes from fosmid *1–19* were amplified by polymerase chain reaction (PCR) using specific oligonucleotides (Table S1 in S1 File). The PCR products were digested with NdeI and NotI (*epux*1) and NheI and XhoI (*epux*2) and separately ligated to pET24a (+) in frame with the vector encoded C-terminal 6x-His tag. The recombinant plasmids were transformed into *E. coli* BL21 (DE3) for overexpression of Epux1 and Epux2.

### Protein expression

Epux1 and Epux2 induction expression was carried out in 1 L of LB medium supplemented with 30 µg mL^-1^ kanamycin. For Epux1, 0.5 mM IPTG (final concentration) was added to the cultures at an Optical Density at 600 nm (OD_600nm_) of approximately 0.6, and the cultures were incubated at 37 °C and 200 rpm for four hours. For Epux2, 0.1 mM IPTG (final concentration) was added at an OD_600nm_ of approximately 1.5, and the cultures were incubated at 20 °C, for 16 hours to promote correct folding and increase its solubility. Cells were harvested by centrifugation (6000 rpm, 10 min, 4 °C) and stored at −80 °C. After defrosting, cells were resuspended in 60 mL of lysis buffer (50 mM NaH_2_PO_4_, 300 mM NaCl, and 10 mM imidazole, pH 8), 1 mg mL^-1^ lysozyme and protease inhibitor cocktail (cOmplete ^™^, Roche) and incubated for 40 minutes on ice. Cells were disrupted by sonication with pulses of 10 s on/ 30 s off, for 20 minutes at 70% amplitude. Cell lysates were clarified by centrifugation (7000 rpm, 1 hour, 4 °C) and used for Impranil clarification assays on agar plates.

### Purification of Epux1 and Epux2

Clarified cell lysate from 1 L of BL21/pET24a-Epux1 and BL21/pET24a-Epux2 culture was mixed with 0.3 mL and 1 mL of equilibrated Ni-NTA agarose matrix (Qiagen, Germany), respectively, and incubated for 12 hours at 10 °C. The matrix was transferred into a gravity flow column and washed with 10 column volumes of wash buffer (50 mM NaH_2_PO_4_, 300 mM NaCl and 30 mM imidazole). Subsequently, Epux1 was eluted with three-column volumes and Epux2 with five-column volumes of elution buffer (50 mM NaH_2_PO_4_, 300 mM NaCl and 100 mM imidazole). The eluted proteins were dialyzed with a 14 kDa cellulose membrane (Sigma-Aldrich) against 50 mM potassium phosphate buffer, pH 7.0, and concentrated by centrifugal ultrafiltration with Amicon Ultra 10K (Millipore). Protein concentration was determined by the Lowry method [26] and purity was evaluated by SDS-PAGE electrophoresis.

A zymographic analysis with the purified fraction of Epux1 was performed to detect the band corresponding to the protein of interest. A tricine semi-native SDS-PAGE was carried out based on the method described by [27] and after electrophoresis, the gel was excised into two fractions. One fraction of the gel was stained with Coomassie, and the other fraction was washed twice with Triton X-100 (2.5%) and incubated in a 50 mM potassium phosphate buffer (pH 7) for 2 hours at 10 °C for renaturing proteins. Subsequently, the gel was placed on top of an agar plate containing 50 mM potassium phosphate buffer (pH 7) and Impranil (0.3%) and incubated at 30 °C for 18 hours.

### Substrate specificity

Enzymatic reactions of 200 µL with 0.4 µM of the purified Epux1 or Epux2 and 0.25 mM of *p*-nitrophenyl esters of different chain lengths (C4, C8, C12, C16, C18) were performed in triplicate in 100 mM KH_2_PO_4_ buffer, pH 7.2. The reactions were set on ice and the release of *p*-nitrophenol was followed for 4 minutes at 30 °C by spectrophotometry at 400 nm in a microplate spectrophotometer UV-Vis. A control

background of autohydrolyzed substrate was considered for all reactions assayed.

Absorbance readings were converted to *p*-nitrophenol concentration using molar extinction coefficient (17,000 M^−1^ cm^−1^) to calculate specific activity for each enzyme. One unit of enzymatic activity was defined as the amount of enzyme that releases 1 µmol of *p*-nitrophenol min ^−1^ at 30 °C.

### Effect of pH and temperature on esterase activity

To evaluate the effect of pH, enzymatic reactions were prepared in Britton-Robinson buffer adjusted at pH values from 5 to 12 as reported by [28]. *p*-nitrophenyl butyrate was used as a substrate. The reactions were performed and the specific activity was calculated as described in the previous section.

For determining the effect of the temperature, the buffer/substrate mixture consisting of p-nitrophenyl butyrate 0.25 mM in 100 mM KH_2_PO_4_ buffer (pH 7.2), was preincubated for three minutes at temperatures from 15°C to 45 °C with increments of 5 °C. After preincubation 0.4 µM of the purified Epux1 or Epux2 were added and the reaction was followed for 4 minutes at each temperature and the relative activity was calculated.

### Qualitative determination of Impranil clarification capacity of purified enzymes

50 µg of each enzyme were added on 1 cm diameter wells in agar plates containing 50 mM potassium phosphate buffer (pH 7.0) and Impranil (0.3%). Plates were incubated overnight at 30 °C to observe clearing halos.

### Enzymatic Impranil degradation reactions

Enzymatic reactions of 1 mL were prepared by adding 1.4 µM of the purified fraction of Epux1 or Epux2 in triplicate to a 1.6 mg mL^-1^ (final concentration) Impranil suspension in 50 mM potassium phosphate buffer (pH 7.0).

Negative controls with Impranil suspension without enzyme and with 1.4 µM of denatured Epux1 or Epux2 by heat (95 °C, 10 min) were used. All reactions were incubated at 30 °C for 18 hours and changes in turbidity were measured spectrophotometrically at OD_600nm_, as an indication of Impranil-DLN hydrolysis [7,29]. Additionally, to test the activity of the enzymes over Impranil with the best conditions of pH and temperature obtained for esterase activity, enzymatic reactions were prepared in Britton-Robinson buffer pH 9 and were incubated at 15 °C for 18 h, and the changes in turbidity were measured spectrophotometrically. A t-test was performed to compare the mean of each treatment and determine its statistical significance with a *p*-value of 0.05.

### FT-IR analysis of enzymatic reactions

After incubation time, the water of the enzymatic reactions described in the previous section was evaporated (37 °C, 5 days). Each reaction was analyzed in an FT-IR spectrometer (PerkinElmer) with an ATR (attenuated total refraction) device. FT-IR spectra were acquired in the range of 400−4000 cm^-1^. Four scans were collected with a resolution of 4 cm^-1^. The background was collected with the empty prism and spectra were analyzed using the Spectragryph v1.2 software [30]. Graphics were obtained using the average of three replicates, according to the Spectragryph automatic algorithm.

### Identification of Impranil degradation products by GC-MS

Liquid-liquid extractions were performed from Impranil enzymatic reactions of 1 mL and controls. 10 mL of dichloromethane (DCM) were added and shaken in a separating funnel for one minute and the organic phase was recovered. This process was performed two times for each reaction. The organic phase was concentrated until 0.5 mL using a reduced pressure evaporator and a nitrogen evaporator.

The extracts were injected into an Agilent 6890N Gas Chromatograph (Santa Clara, CA, USA), using an HP-5 245 column. The oven temperature was set at 60°C and increased 3°C per minute to 220°C. The injector temperature was set at 250°C. Helium was used as the carrier gas at 1 mL ⋅ min^-1^. Electron impact mass spectra were collected at 70 eV in the range of m/z 30–247. Ion source and analyzer temperatures were set at 250 and 230 °C, respectively. The NIST V.11 mass spectral library was used for compound identification. Compounds with mass spectral similarity values close to 70 or higher were considered the same compounds as those in the library [7].

## Results and discussion

### Screening for esterase and Impranil-clearing activity

A fosmid metagenomic library of 40,000 CFUs was constructed with eDNA from sediments of the Apatlaco River (library size = 1.6 Gb; clone insert size ~40 kb). Since the main enzymatic activity related to PU biodegradation is ester hydrolysis [6,7], we performed functional screenings using tributyrin as a substrate. On the other hand, we used the commercial polyester-PU suspension Impranil to detect PU degrading activity [29,31,32], as it can be considered as a model substrate to study polyester-PU biodegradation. Of 941 evaluated clones, we found four positives after the double screening assay using tributyrin and Impranil substrates in agar plates, where clones with degradation halos were selected. The reduced hit rate of impranil-positive (1:235) clones compared to those with tributyrin (1:17) was somehow expected because of the complexity difference between both substrates, similar to previous reports for Impranil and long-chain compounds [33,34]

To date, a few PU-degrading enzymes have been isolated from metagenomic libraries by tributyrin-based screenings [35,36], and their activity was tested based on their close sequence relationship to other experimentally validated bacterial enzymes using specific PU compounds. However, functional metagenomics based on PU-type substrates could lead to identifying PU-degrading enzymes different from those previously described. For example [33], identified an Impranil-degrading carboxylesterase (CE_Ubrb) from a metagenomic library through a primary screening on Tween 20 and a secondary screening on Impranil. Additionally, three urethanases, were isolated through functional metagenomics from a library constructed from soil PU-contaminated samples by a high throughput proprietary screening [37]. These enzymes showed activity on dicarbamates from glycolysis of polyether polyurethane.

### DNA sequencing and analysis

**Fig 1:**
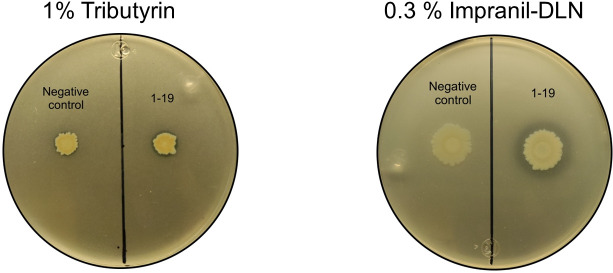
*E. coli* clone *1–19* showing lipolytic and clearing Impranil activity. *E. coli* clone *1–19* in LB agar + tributyrin and LB agar + Impranil-DLN, and negative control (*E. coli* Epi300/PCC2FOS-control insert) incubated for three days at 37 °C and one week at 30 °C.

From the four clones with activity on lmpranil, we selected the clone that showed the largest degradation halo in a shorter incubation period for further characterization. The *1–19* fosmid DNA (Fig 1) was isolated and sequenced to identify genes potentially involved in PU biodegradation. The fosmid fragment size was ~ 46 kb and shared 99% sequence identity with a fragment from a complete genome of *Acinetobacter sp.* NEB 394 (accession: CP055277.1), consistent with the high relative abundance of this genus previously reported in the Apatlaco River [17]. We predicted 44 ORFs of which two corresponded to genes encoding enzymes of the α/β hydrolase superfamily that we annotated as Epux1 (1014 bp), and Epux2 (1251 bp), respectively. Most of the known PU-degrading enzymes belong to this superfamily, which includes esterases, lipases and proteases.

Epux1 contains a putative BD-FAE (Bifunctional feruloyl and acetyl xylan esterase) domain and a signal peptide sequence predicted by SignalP [38] (Fig S1 in S1 File), which indicates that the protein is exported extracellularly. Epux2 contains a predicted *β*-lactamase domain, which has not been previously associated with a PU-degrading function. Both Epux1 and Epux2 share < 30% sequence identity with other characterized carboxylesterases. These two genes were located distantly from each other and do not belong to an operon.

### Phylogenetic analysis

**Fig 2:**
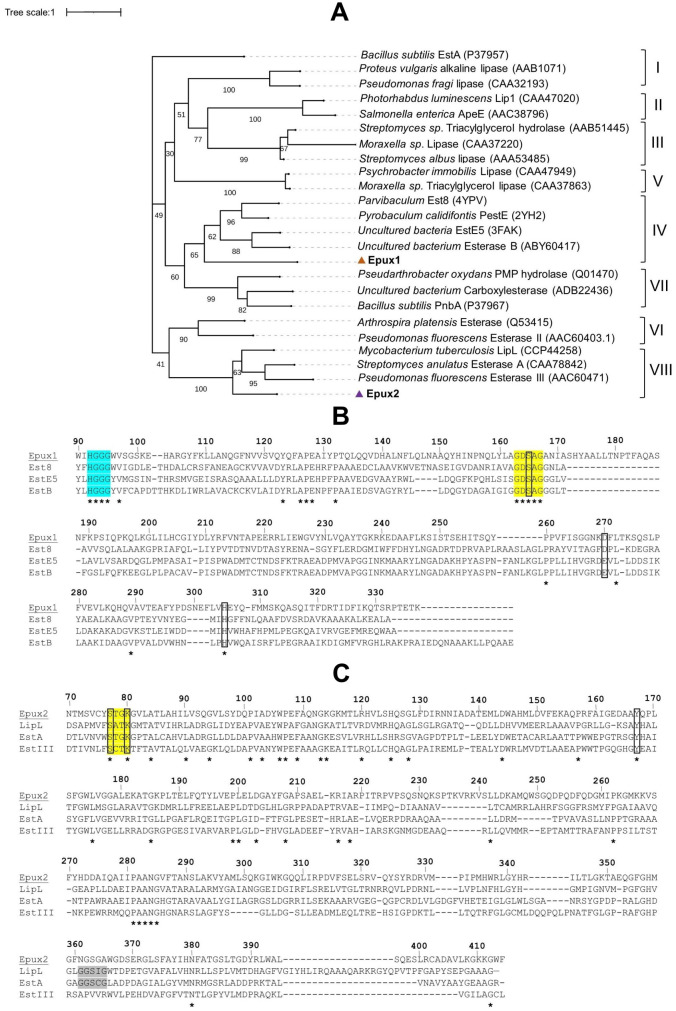
Phylogenetic analysis and sequence alignments of Epux1 and Epux2 with other lypolitic enzymes. A) phylogenetic analysis of Epux1 and Epux2 and 22 characterized lipolytic enzymes representing eight different families. B) Amino acid sequence alignment of Epux1 and other family IV carboxylesterases. Est8 (PDB: 4YPV), EstE5(PDB: 3FAK), and EstB (GenBank: ABY60417). Characteristic motifs for family IV are colored: oxyanion hole in cyan and pentapeptide GDSAG in yellow. C) Amino acid sequence alignment of Epux2 and other family VIII carboxylesterases. LipL (GenBank: P71778), EstA (GenBank: CAA78842), and EstIII (GenBank: AAC60471). The characteristic motif for family VIII is colored in yellow and the GXSXG motif in LipL and EstA is colored in gray. Asterisks indicate fully conserved amino acids, and amino acids that form the catalytic triad are indicated by boxes.

To establish the evolutionary relationship of Epux1 and Epux2 with other carboxylesterases, a phylogenetic tree was constructed with the amino acid sequences of 22 characterized bacterial lipolytic enzymes representatives of 8 different families, according to the classification proposed by [39] (Fig 2A). Epux1, which contains a conserved pentapeptide GDSAG (Fig 2B), was clustered along with family IV carboxylesterases, from which several enzymes have been described as substrate-promiscuous [40,41], being capable of hydrolyzing more than 60 different carboxylic esters. Such substrate promiscuity has been associated with the flexibility of the active site cavity and the ratio of the cavity volume and solvent-accessible surface area.

Epux2 was clustered with members of family VIII carboxylesterases, which share the lactamase/peptidase conserved motif SXXK, containing the nucleophilic serine [42,43], although some enzymes of this family could also contain a pentapeptide GXSXG [44] (Fig 2C).

### Structure prediction of Epux1 and Epux2

We generated *de novo* models for Epux1 and Epux2 through AlphaFold3 to identify features in their three-dimensional structures that could favor the interaction with bulky substrates like PU.

The quality of the models was validated using the SAVES v6.0 server. The Ramachandran plot values showed that 91.7% and 90.8% of the residues in the models of Epux1 and Epux2, respectively, were found in the most favorable region. Additionally, the ERRAT test values were 99.08 for Epux1 and 99.55 for Epux2, showing that both models are reliable for further analysis.

**Fig 3:**
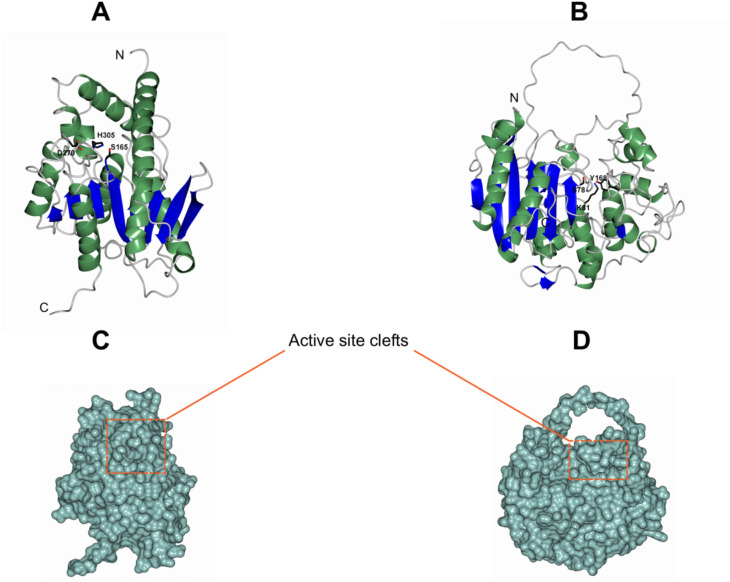
3D structural models of Epux1 and Epux2. A and B, ribbon representation of the folding of Epux1 and Epux2, respectively. C and D, surface representation of Epux1 and Epux2, respectively. The active site cleft in each protein is indicated by a dotted rectangle. All figures are based on AlphaFold models.

According to the models, both enzymes share an α/β core domain but differ in their global architecture (Fig 3A–D). A search in the Dali protein structure comparison server [45], showed that the closest structural homolog for Epux1 is the family IV carboxylesterase Est8 (PDB ID 4YPV) [46], with a root mean square deviation (rmsd) of 2.6 Å, and 21% of sequence identity. The structure of this enzyme, as well as that of Epux1, consists of an α/β domain composed of a twisted β-sheet of mostly parallel strands surrounded by α-helices and a small helical domain that covers the active site, known as the cap domain (Fig 3A). The function of the cap domain is related to substrate pre-recognition [40], and its arrangement determines the substrate accessibility to the active site entrance [46,47]. The mobility of the cap domain has been proposed as an important feature for substrate promiscuity of family IV carboxylesterases [48].

A comparison of Epux1 to Est8 and other structural homologs (EstD11; PDB ID 7AT0 [47], and EstE5; PDB ID 3FAK [49]) shows that the configuration of the cap domain differs in Epux1 due to the absence of an α-helix close to the active site, which is conserved in all the other compared enzymes (Fig S2 in S1 File). This feature could make the active site cavity of Epux1 comparatively more accessible, favoring its interaction with bulky substrates such as PU. By superposition with Est8, the putative catalytic triad (Ser^165^, Asp^270^, His^305^) and the conserved motif ^92^HGGG^95^, which forms the oxyanion hole in Est8 and other family IV members, were identified for Epux1 (Fig S3 in S1 File).

Epux2 has a structure similar to class C β-lactamases, consisting of an α/β domain composed of a β-sheet of antiparallel strands flanked by α-helices and a small helical domain where the active site is located. Its closest structural homolog is the family VIII carboxylesterase EstSRT1 (PDB: 5GMX) with an rmsd of 2.1 Å and 29% sequence identity, according to the Dali server. EstSRT1 showed hydrolytic activity toward bulky cephalosporins, which the authors associated with the flexibility of the active site [50]. The putative catalytic triad in Epux2 was identified by superposition with EstSRT1, consisting of residues S^78^, K^81^, and Y^168^ (Fig S4 in S1 File).

Another feature in Epux2 structure is the presence of a loop (Ω loop) over the active site entrance, which is conserved in some family VIII carboxylesterases and β-lactamases (Fig 3B, D). The length of this loop varies in length and has been related to substrate specificity [44,51,52]. Although for Epux2 the Ω loop was modeled with low confidence, according to the pLDDT scores in this region (50–70), it appears to be longer than that of EstSRT1.

Despite the differences in the tridimensional structure of Epux1 and Epux2, the comparison of each one with their closest structural homologs shows the potential of these enzymes to interact with bulky substrates. However, to corroborate this hypothesis further studies are needed, including experimental structure analyses and mutational studies, that could offer a clearer understanding of the residues involved in substrate interaction.

### Expression and purification of Epux1 and Epux2

The genes coding for Epux1 and Epux2 were expressed separately to determine whether the enzymes could hydrolyze tributyrin and PU. Both enzymes were expressed in *E. coli* with a C-terminal 6xHis tag for affinity chromatography purification. Additionally, Epux1 was expressed along with its native N-terminal signal peptide.

Although Epux1 was expressed at low levels and not fully soluble, it was partially purified with a yield of 0.28 mg/L. SDS–PAGE analysis of the purified fraction revealed the presence of three bands with close molecular weights between 30 and 36 kDa (Fig S5 in S1 File). To identify the protein band with the enzymatic activity, a zymogram analysis was performed using Impranil as the substrate. A degradation halo zone was observed for a protein with an approximate molecular weight of 36 kDa (Fig S5 in S1 File), which corresponds to the calculated molecular weight of Epux1 without the processed signal peptide. The signal peptide of Epux1 is classified as sec/SPII according to SignalP 6.0 (https://services.healthtech.dtu.dk/services/SignalP-6.0/) (Fig S1 in S1 File) and could be recognized and processed by *E. coli,* as reported for other heterologously expressed proteins [53].

**Fig 4:**
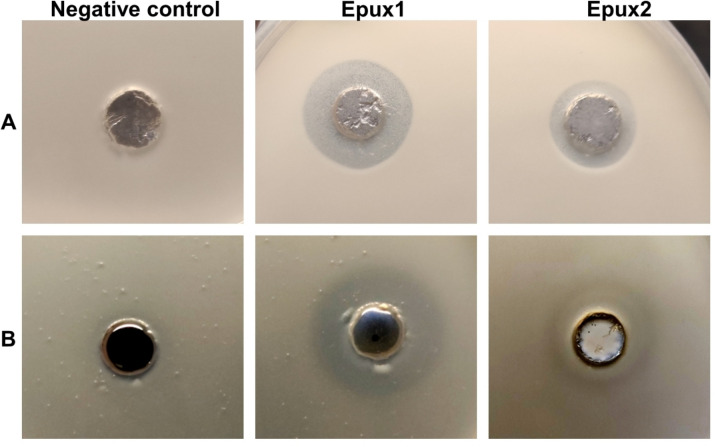
Enzymatic clearing activity of Epux1 and Epux2 on tributyrin and Impranil. Enzymatic clearing assays of Epux1 and Epux2 on agar containing A) 1% tributyrin, B) 0.3% Impranil. Negative control: *E.coli*/BL21 pET24a cell extract.

On the other hand, Epux2 (47.7 kDa) was expressed at high levels in *E. coli* and was successfully purified, as confirmed by SDS page electrophoresis (Fig S6 in S1 File), with a yield of 27.9 mg/L. The activity of both enzymes was tested in agar plates added with tributyrin and Impranil, where both exhibited clearing halos in both substrates (Fig 4). However, Epux1 seems to be more effective on Impranil, showing a larger and clearer halo compared to Epux2 (Fig 4B). This weaker activity of Epux2 could be explained by a lower solubility, as protein precipitation was observed after incubation in the well where it was applied.

### Substrate specificity

**Fig 5:**
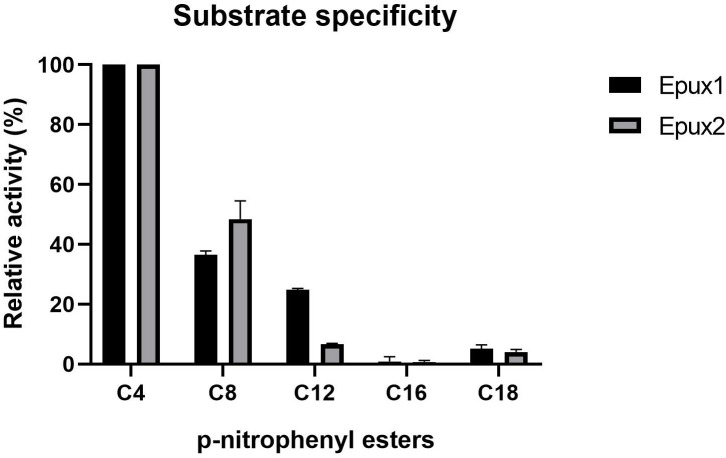
Substrate specificity of Epux1 and Epux2 against *p*-nitrophenyl esters. The enzymatic activity was determined at 30 °C, in KH_2_PO_4_ buffer (pH 7.2) using *p*NP-butyrate (C4), *p*NP-caprylate (C8), *p*NP-laurate (C12), *p*NP-palmitate (C16), or *p*NP-stearate (C18) as a substrate. The average values of three replicates are shown together with error bars.

We measured Epux1 and Epux2 activity through spectrophotometric analysis using *p*-nitrophenyl esters of different chain lengths (C4 - C18) as substrates. Both enzymes exhibited the highest activity against a short chain substrate (C4), however, both were active on a medium chain substrate (C8) and Epux1 showed more than 20% of relative activity against the C12 substrate (Fig 5). These results are consistent with the wide substrate specificity reported for family IV carboxylesterases. Enzymes from family IV have shown great versatility, having the ability to hydrolyze different kinds of esters, from short-chain acyl esters [46,49] to aromatic esters [40,47]. Some family VIII carboxylesterases, which share sequence and structure similarity with class C *β*-lactamases, can hydrolyze not only ester substrates but also *β*-lactams, such as penicillin and cephalosporin derivatives [43,44,50]. However, it has been shown that some enzymes act exclusively on the ester bonds of *β*-lactams and not on the amide bonds; this difference in reactivity could be explained by steric reasons [42].

### Optimal pH and temperature

**Fig 6:**
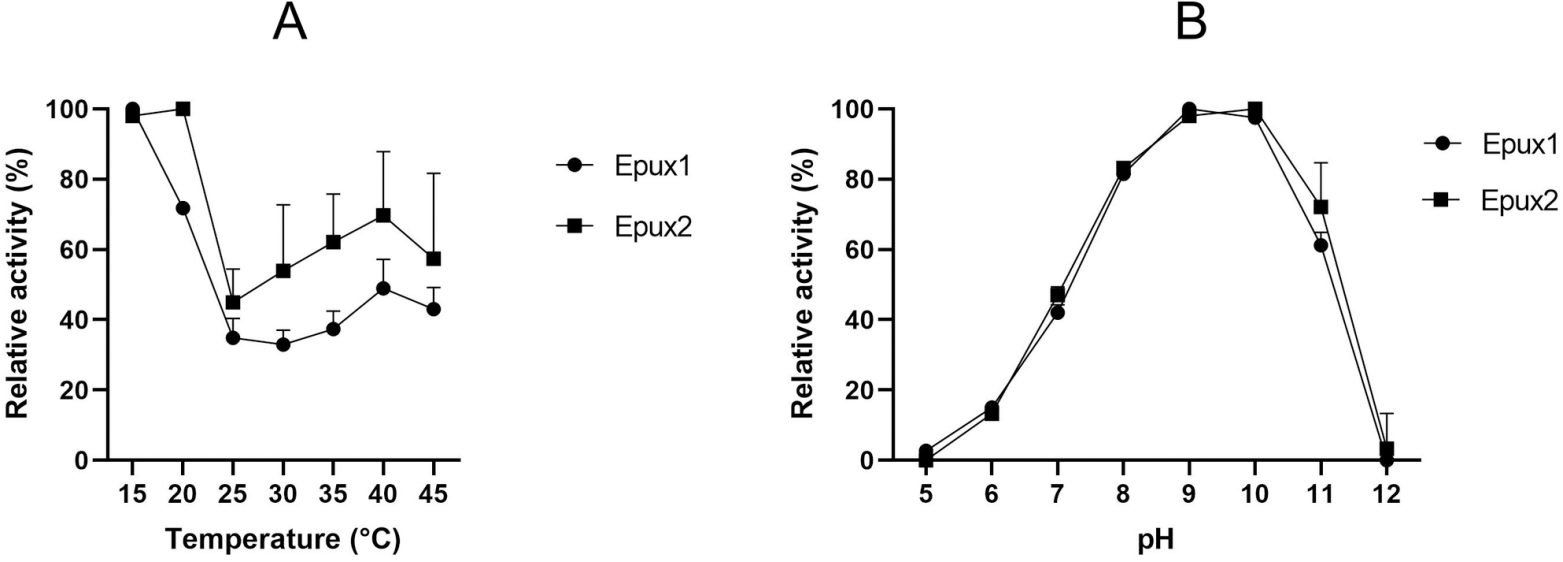
Effect of temperature (A) and pH (B) on esterase activity of Epux1 and Epux2 with *p*-nitrophenyl butyrate (C4) as substrate. The average values of three replicates are shown together with error bars.

To determine the optimal conditions of pH and temperature for each enzyme, we performed enzymatic reactions with *p*-nitrophenyl butyrate using different temperatures and pH values. Both enzymes were highly active at low temperatures, with the maximum relative activity observed at 15 °C, for Epux1 and at 15–20 °C for Epux2. However, both enzymes maintain at least 40% of relative activity in the range of 25–45 °C (Fig 6A). The enzymes showed optimal relative activity under alkaline conditions (pH 9 and 10), being active under a wide range of pH (from 6 to 11) (Fig 6B). These results showed the versatility of conditions under which these enzymes can work, thereby signifying their suitability for industrial applications. Concerning the origin of the enzymes, especially Epux1, which is an extracellular enzyme, it could be hypothesized that the enzyme is able to work under diverse environmental conditions.

### Impranil clearing activity

**Fig 7:**
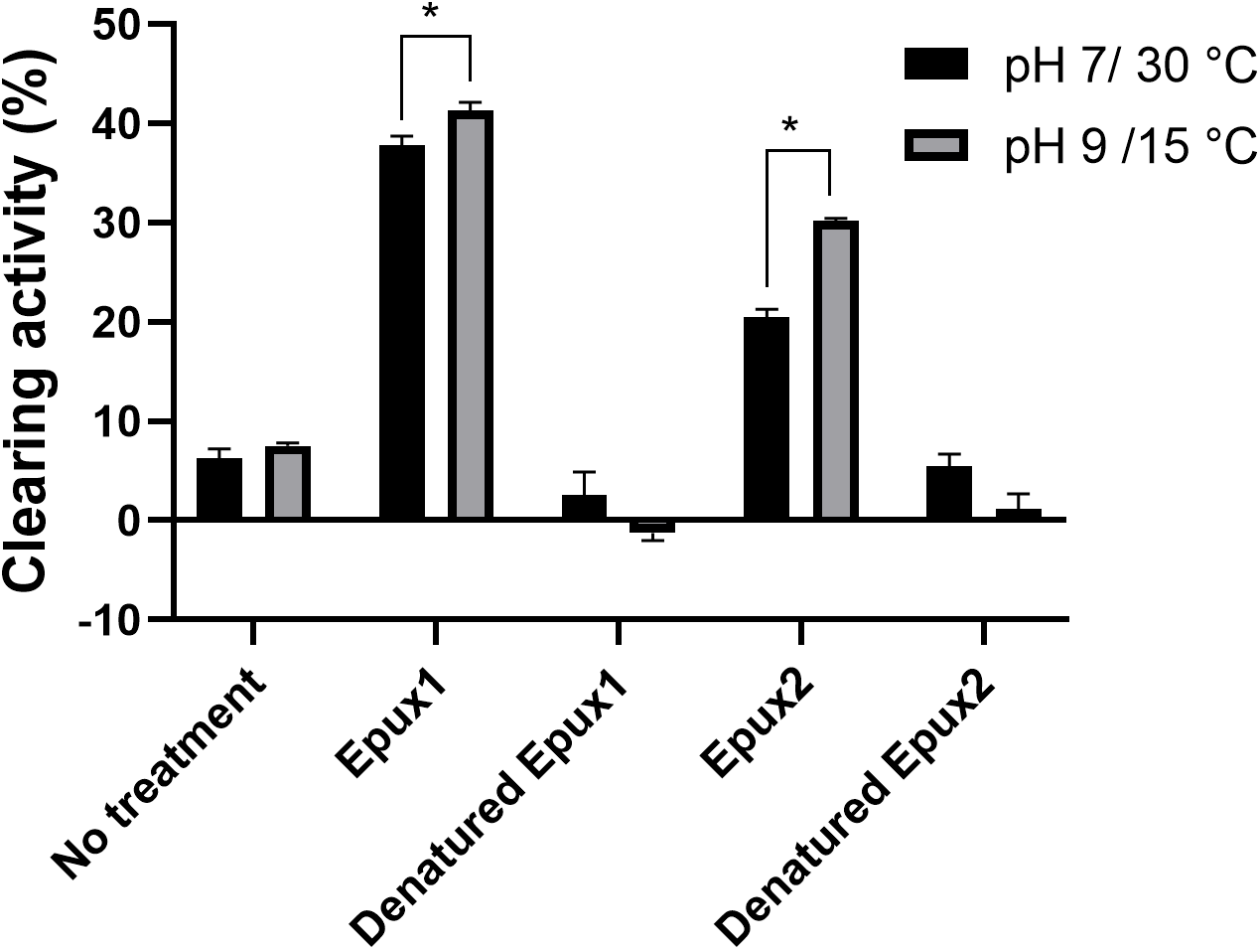
Impranil clearing activity of Epux1 and Epux2 in different conditions of pH and temperature. Reactions of 1 mL containing Impranil (1.6 mg/mL) in potassium sodium buffer (pH 7) or Britton-Robinson buffer (pH 9). Denatured Epux1 and Epux2 and Impranil suspension without enzyme were used as negative controls. Each graphic represents the average of three replicates ± standard deviation, * significant difference (α = 0.05).

To further demonstrate the activity of Epux1 and Epux2 on PU, we measured the clearance of Impranil by turbidimetric analysis. Each enzyme was added to an Impranil suspension and incubated for 18 hours. We compared the clearing activity under two conditions: pH 7, 30°C (standard, reported for other PU-degrading enzymes) and pH 9, 15°C (optimal conditions established in this work for esterase activity) (Fig 7).

Epux1 exhibited a similar clearing activity in both tested conditions, being better at pH 9, 15 °C (37 and 41%, respectively), while Epux2 showed a higher clearing activity under pH 9 and 15 °C (30%) compared to pH 7 and 30 °C (20%). These assays confirm the capability of Epux1 and Epux2 to work under different conditions of pH and temperature. Clearing activity was not observed or barely detected in the negative controls (denatured Epux1 or Epux2 and Impranil suspension without enzymatic treatment) (Fig 7). As shown in Table 1, optimal temperatures of previously reported PU-degrading enzymes are above 30 °C, but Epux1 and Epux2 are the first reported PU-degrading enzymes able to work at low temperatures.

**Table 1:** Reported PU-degrading enzymes and their properties.

Enzyme	Strain	Substrates	Optimal temp/ph	Clasification	References
**Epux1**	*Acinetobacter sp.* *(*metagenomic)	*p*-nitrophenyl butyrate *p*-nitrophenyl octanoate *p*-nitrophenyl dodecanoate Impranil-DLN	15 °C/9	Esterase	This work
**Epux2**	*Acinetobacter sp.* (metagenomic)	*p*-nitrophenyl butyrate *p*-nitrophenyl octanoate Impranil-DLN	15-20 °C/9	Esterase	This work
**Polyurethane esterase (PudA)**	*Comamonas acidovorans*	Tributytirin Polylactic acid	45°C/ 6.5	Esterase	[54]
**Polyurethanase A (PueA)**	*Pseudomonas chlororaphis*	*p*-nitrophenyl acetate	ND	Esterase/Lipase	[55]
**Polyurethanase B (PueB)**	*Pseudomonas chlororaphis*	*p*-nitrophenyl butyrate *p*-nitrophenyl caproate *p*-nitrophenyl caprilate	30°C	Esterase/Lipase	[56]
**Polyester hydrolase (PE-H)**	*Pseudomonas aestusnigri*	*p*-nitrophenyl butyrate BHET PET	30°/ 7.4	Polyester hydrolase	[57]
**Polyurethanase**	*Acinetobacter gerneri*	Impranil-DLN *p*-nitrophenyl propionate	55°C/ 10	Esterase	[58]
**Cutinase (Tfcut2)**	*Thermobifida fusca*	*p*-nitrophenyl butyrate Tributyrin Impranil-DLN	70° C/ 8	Cutinase	[32]
**Cutinase (LCC)**	Metagenomic	*p*-nitrophenyl butyrate PET Impranil-DLN	50°C/ 8.5	Cutinase	[32,59]
**Cutinase (Tcur0390)**	*Thermomonospora Curvata*	*p*-nitrophenyl butyrate PET Impranil-DLN	55°C/ 8.5	Cutinase	[32,60]
**Cutinase (Tcur1278)**	*Thermomonospora Curvata*	*p*-nitrophenyl butyrate PET Impranil-DLN	60°C/ 8.5	Cutinase	[32,60]
**Urethane hydrolase**	*Rhodococcus equi*	*p*-nitrophenyl butyrate Urethane	45°C/ 5.5	Amidase/ esterase	[54]
**Oxidoreductase (Oxr-1)**	*Bacillus velezensis*	PU emulsion	ND	Oxidoreductase	[61]
**Amidase (GatA250)**	*NR*	*p*-nitrophenethyl-acetamide *p*-nitrophenyl butyrate PU foam and film	30°C/ 7–8	Amidase	[62]
**Hfor_PE-H**	*Halopseudomonas formosensis*	Impranil-DLN *p*-nitrophenyl hexanoate	50 °C/ 7.2	Polyester hydrolase	[8]
**UMG-SP1**	Metagenomic	7-carbethoxy-4-methylcoumarin Low molecular weight dicarbamates	70 °C/ 10	Amidase	[37]
**UMG-SP2**			70 °C/ 10		
**UMG-SP3**			35 °C/ 10		

ND: not described.

Clearing activity on Impranil has been directly associated with PU-degrading activity, being reported for crude enzymatic extracts [6,7] and purified enzymes [6,32] reported a decrease of 25 A_600_ units/min/mL of enzyme with an esterase in crude extract in reactions of 5 minutes [29] reported a decrease in A_600_ of 0.53 ± 0.07 in reactions of 24 h using 7 μg/mL of a commercial *Pseudomonas sp* Lipase. In the same study, an esterase from *Pseudomonas fluorescens* showed no clearing effect while a protease from *Bacillus sp.* had a clearing effect due to Impranil aggregation. It is important to mention that there was no apparent Impranil aggregation in Epux1 or Epux2 reactions. Given the different parameters and methods utilized to calculate clearing activity on Impranil it is difficult to make a direct comparison between the different enzymes reported.

### Analysis of Impranil biodegradation by FT-IR spectroscopy

**Fig 8:**
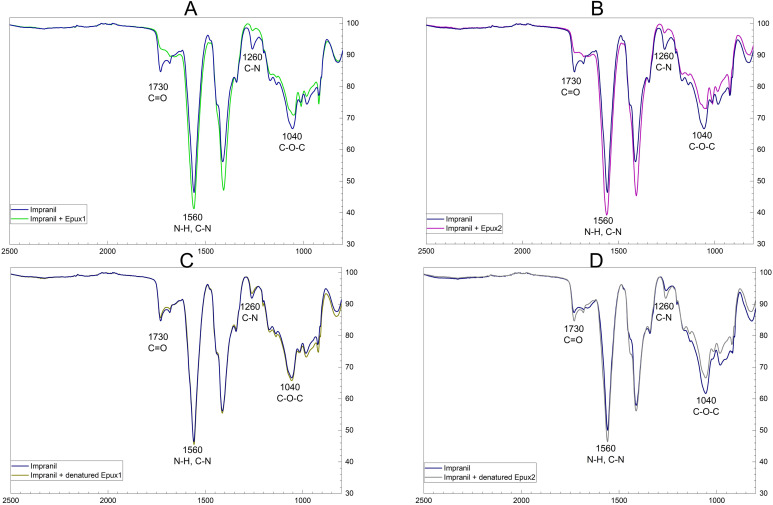
FT-IR spectra comparison between Impranil suspension without enzymatic treatment and treatment with Epux1 (A) and Epux2 (B), and the controls with denatured Epux1 (C) or denatured Epux2 (D). Impranil suspension without enzymatic treatment (blue), treated with Epux1 (green) and with Epux2 (magenta). As negative controls denatured Epux1 (brown) and Epux2 (clear gray) were utilized. Reactions of 1 mL; 1.6 mg/mL Impranil in Britton-Robinson buffer (pH 9), with 1.4 μM of Epux1 or Epux2, incubated 18 h at 15 °C. Each graphic represents the average of three replicates obtained by Spectragryph.

The above results demonstrate the esterase activity of the recombinant Epux1 and Epux2 and suggest their activity on polyester-PU. To determine the chemical changes that occurred in the polymer due to the action of Epux1 and Epux2, the *in vitro* enzymatic reactions were dried and analyzed by FT-IR spectroscopy. The treatment with each one of the enzymes decreased considerably the signal of the carbonyl group, C = O (1730 cm^-1^), which indicates the breaking of ester bonds. In addition, they generated an increase in the N-H and C-N signal (1560 cm^-1^). This could be an indication of the releasing of these groups from the molecule. Conversely, the changes in the C-O-C and C-N signals (1040 and 1260 cm^-1^, respectively) are not consistent, because they are also observed in the negative controls of the denatured Epux2, but not in the denatured Epux1 (Figs 8A–D, Table S2 in S1 File).

The main changes reported in the biodegradation analyses of PU (including Impranil) using FT-IR spectroscopy are the decrease in the C = O stretching vibrations (1730 cm^-^1); related to the ester and urethane groups, the N-H and C-N stretch signal (1560 cm^-1^); related to urea and urethane, and the C-O-C stretch in 1040 cm^-1^, attributable to urethane or ester groups [7,63,64]. In the cited studies, the aforementioned spectroscopic changes were induced by combined enzymatic activities from fungi or bacteria. However, there are few reports of FT-IR spectroscopy of enzymatic biodegradation with purified enzymes [32] reported changes in the carbonyl sign and in the C-O-C stretching using a cutinase. In addition to the changes in the carbonyl sign [29], detected changes in the peaks at 1540 cm^-1^ and 1400 cm^-1^, which were attributed to the carboxylate stretches as a product of the polymer hydrolysis by a lipase enzyme.

The FT-IR data in our analysis suggest that Epux1 and Epux2 can hydrolyze the polyester PU in Impranil, being capable of hydrolyzing the ester bonds in the polymer. However, further experiments are needed to determine the specific type of bonds that each enzyme breaks in the polymer.

The combination of enzymatic clarification assays and FT-IR spectroscopy led us to confirm the activity of the Epux1 and Epux2 on polyester PU. The use of appropriate controls was useful to detect the relevant changes in the signals that could be associated with enzymatic activity, rather than changes caused just by the presence of the enzyme in the mixture.

### Analysis of Impranil biodegradation by GC-MS

**Fig 9:**
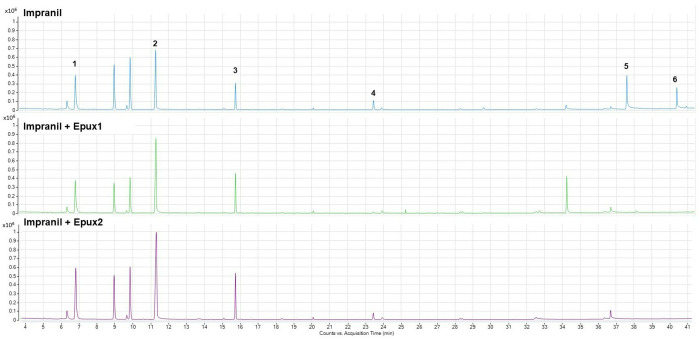
GC-MS chromatograms of the DCM extracts of Impranil-DLN suspensions treated with Epux1 or Epux2 and the negative control (Impranil-DLN suspension without enzymatic treatment). Reactions of 1 mL; 1.6 mg/mL Impranil in Britton-Robinson buffer (pH 9), with 1.4 μM of Epux1 or Epux2, incubated 18 h at 15 °C.

With the purpose of identifying products from the enzymatic degradation of polyurethane, the Impranil suspensions treated with each enzyme and the negative control (Impranil suspension without enzymatic treatment) were analyzed by GC-MS. A comparison of the negative control with the enzymatic treatments enabled the identification of the increase in the peak areas corresponding to the building blocks of the polymer, which have been reported as products of biodegradation [7,8] (Table 2). These products are 1,6-hexanediol, neopentyl glycol (only increased in Epux2 treatment), and hexamethylene diisocyanate, which can be released from the polymer by esterase enzymatic activity. On the other hand, the peak areas that correspond to adipic acid derivatives decreased with the enzymatic treatment being undetectable in some cases (Fig 9). According to the NIST library, these products share similarity with adipic acid esters, which could be hydrolyzed by the enzymes. These results are consistent with the FT-IR data, and confirm the capacity of the enzymes to break down the polymer releasing its building blocks.

**Table 2:** Compounds from Impranil-DLN enzymatic treatment with Epux1 and Epux2 detected by GC-MS. The areas represent the average of three replicates.

Peak	Compound	R.T. (min)	Area		
			Impranil	Impranil + Epux1	Impranil + Epux2
**1**	Neopentyl glycol	6.79	4.44E + 06	3.85E + 06	5.89E + 06
**2**	1,6- Hexanediol	11.27	6.30E + 06	9.08E + 06	1.66E + 07
**3**	Hexamethylene diisocyanate	15.73	9.59E + 05	1.66E + 06	2.38E + 06
**4**	Adipic acid derivatives	23.43	3.85E + 05	n.d.	2.93E + 05
**5**		37.58	2.10E + 06	n.d.	n.d.
**6**		40.38	1.23E + 06	n.d.	n.d.

n.d.: Not detected.

Impranil-DLN can be considered as a model substrate and has led to the discovery of several enzymes as Epux1 and Epux2 with potential for being applied in processes of PU bio recycling. However it is necessary to test these enzymes on PU residues from commercial products to determine their real efficiency and applicability. Immobilization of enzymes could increase their applicability in industrial processes for PU upcycling. Although this approach has not yet been reported for PU, it has been studied for the depolymerization of PET [65] and polycaprolactone [66], resulting in an increase in stability and activity of the enzymes.

## Conclusion

This study reports the discovery of two novel bacterial esterase enzymes, belonging to family IV and VIII carboxylesterases, derived from a metagenomic library of a polluted river. As mentioned, a polluted environment such as that found in the Apatlaco River exerts a selection pressure over microorganisms to degrade contaminants and use them as a carbon source. This is encoded in the metabolic potential that can be explored through eDNA and metagenomics. We have demonstrated that a fosmid library screening is a good mining approach to explore the metagenomic information to find enzymes with bioremediation potential. The Epux1 and Epux2 enzymes showed clearing activity on a polyester PU resin, and by spectroscopic FT-IR and GC-MS analysis its degradation activity was confirmed, which we propose to be mediated by the hydrolysis of ester bonds in the polymer. The capacity of the enzymes to interact with complex substrates like polyester PU Impranil, could be associated with its predicted active site accessibility observed in their 3D structures. This interaction capability is an important feature in other plastic-degrading enzymes.

The ability of Epux1 and Epux2 to be active at low temperatures makes them interesting biocatalysts for industrial applications. Cold-active enzymes are advantageous in terms of energy savings and because carrying out the processes at low temperatures prevent unwanted side reactions or degradation of thermosensitive products [67].

The discovery of these novel esterases contributes to the understanding of plastic enzymatic degradation and reflects the potential of eDNA as a source of biocatalysts for bioremediation.

## Supporting information

S1 File‌ (PDF)

S1 Raw images (PDF)

S1 Fig Striking-image. (TIF)
