# Supplementary material for: Enzymatic characterization and polyurethane biodegradation assay of two novel esterases isolated from a polluted river
Source: PLoS One. 2025 Jul 23;20(7):e0327637. doi: 10.1371/journal.pone.0327637 (PMC12286390; doi:10.1371/journal.pone.0327637)
Supplement: S1 File‌ — (PDF) [file pone.0327637.s001.pdf]

Supporting information

Enzymatic characterization and polyurethane biodegradation assay of two novel esterases isolated from a polluted river

Arianna Soto-Hernandez [1], Luis Felipe Muriel-Millan[1a], Adolfo Gracia[2], Alejandro Sanchez-Flores[3] and Liliana Pardo-Lopez [1*]

[1] Departamento de Microbiología Molecular, Instituto de Biotecnología, Universidad Nacional Autónoma de México. Cuernavaca, Morelos, México.

[1a] Departamento de Microbiología Molecular, Instituto de Biotecnología, Universidad Nacional Autónoma de México, Cuernavaca, Morelos, México. Present address: Centre d'Infection et d'Immunité de Lille, Institut Pasteur de Lille. Lille, France.

[2]Instituto de Ciencias del Mar y Limnología, Universidad Nacional Autónoma de México, Coyoacán, México.

[3]Unidad Universitaria de Secuenciación Masiva y Bioinformática, Universidad Nacional Autónoma de México, Cuernavaca, Morelos, México.

***Corresponding author:** e-mail: liliana.pardo@ibt.unam.mx

Table S1. List of primers used for amplification of *epux1* and *epux2* from fosmid 1-19. *Restriction site sequences are underlined.

Gene	Primer direction	Primer sequence	Restriction site*
epux1	**Forward**	5' -GATT<u>CATATG</u>ATTTTTCAATCAATAATAAAAGTACT TAGTACTGTTTTC -3'	*NdeI*
epux1	**Reverse**	5'-GATT<u>GCGGCCGC</u>TTTTGTTTCAGTCGGCCTTGAG GT - 3'	*NotI*
epux2	**Forward**	5'-GATT<u>GCTAGC</u>ATGAAAATTTTAAAACAATATTTACA AGCGAATCC - 3'	*NheI*
epux2	**Reverse**	5'-GATT<u>CTCGAG</u>AAACCAGCCTTTTTTGCCTTTTA AAAC – 3'	*XhoI*

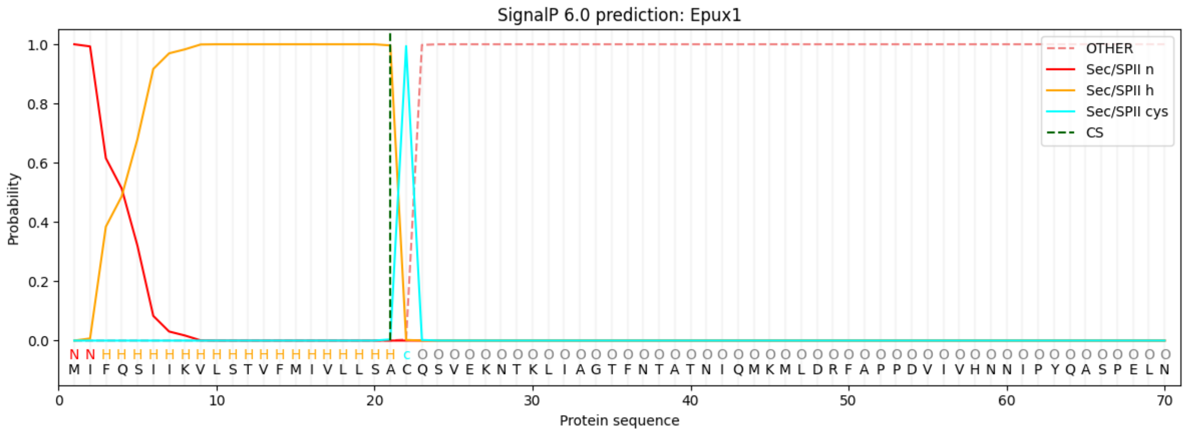

Figure S1. Signal peptide prediction in Epux1 sequence from SignalP 6.0 server (https://services.healthtech.dtu.dk/).

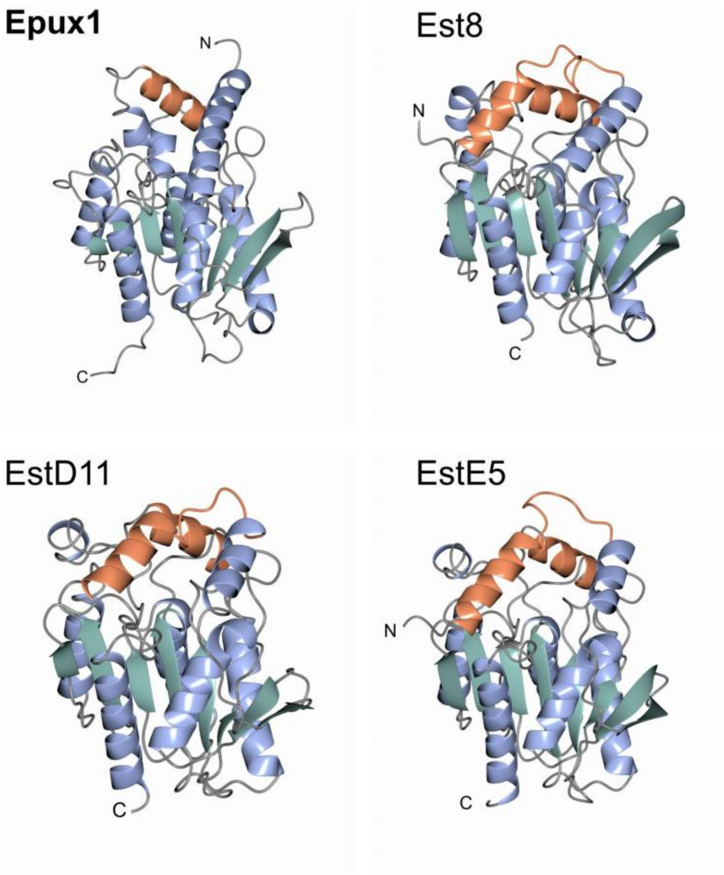

Figure S2. Comparison of cap domains in Epux1 and other family IV carboxylesterases. Est8 (PDB: 4YPV), EstD11 (PDB: 7AT0), EstE5 (PDB: 3FAK). The α/β domain is colored in blue and the cap domain is colored in coral.

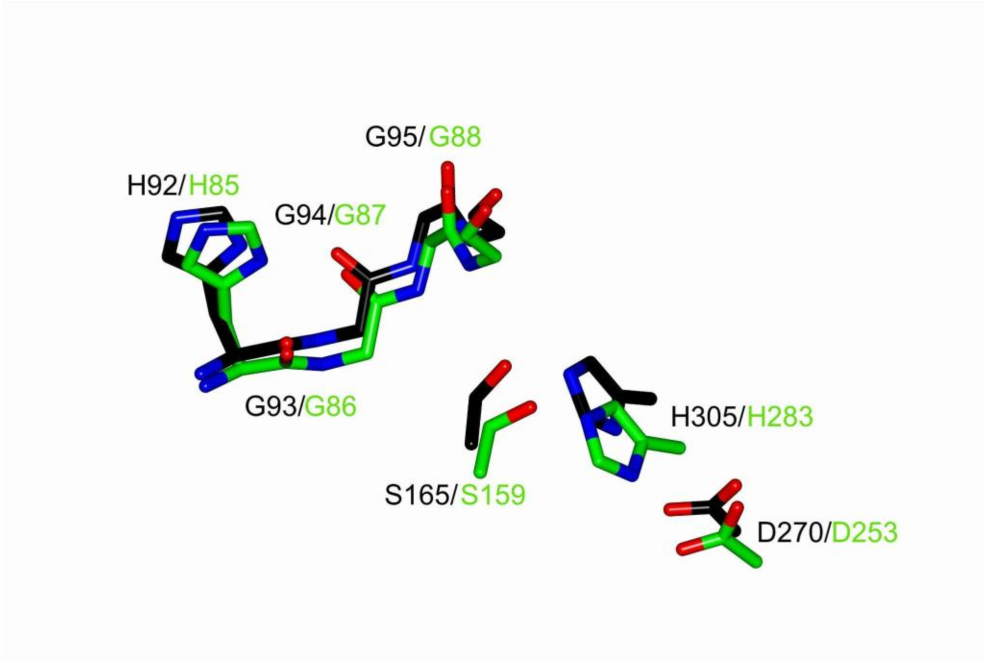

Figure S3. Superimposition of the catalytic triad and HGGG motifs in Epux1 and Est8. Black legends and black carbon backbone correspond to Epux1. Green legends and green backbone correspond to Est8 (PDB: 4YPV).

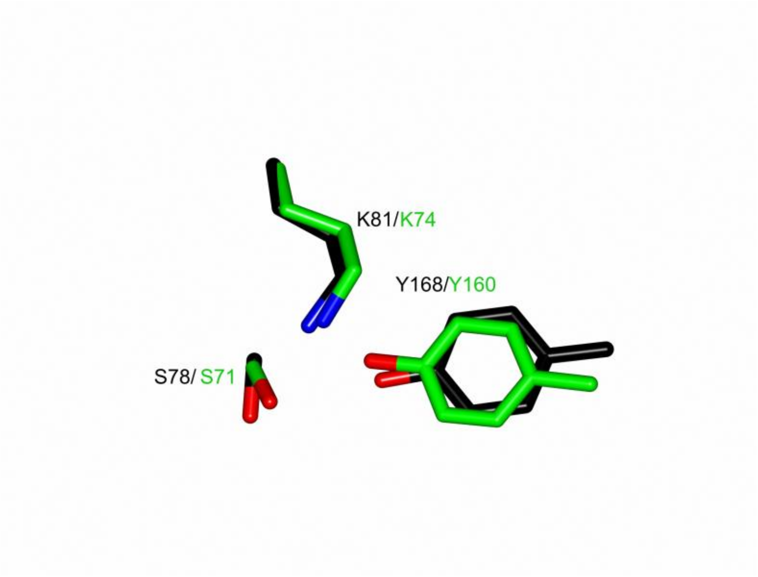

Figure S4. Superimposition of the catalytic triad in Epux2 and EstSRT1. Black legends and black carbon backbone correspond to Epux2. Green legends and green backbone correspond to EstSRT1 (PDB: 5GMX).

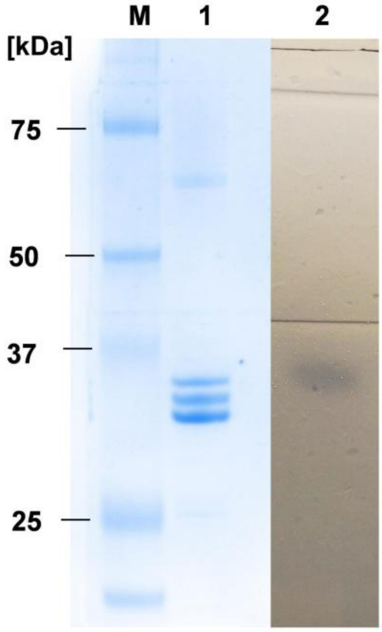

Figure S5. Semi-native SDS–PAGE and zymogram analysis of Epux1. Lane **M**: standard protein marker, lane **1**: purified fraction of Epux1, lane **2**: activity of Epux1 on an agar plate containing Impranil (0.3 %) as the substrate.

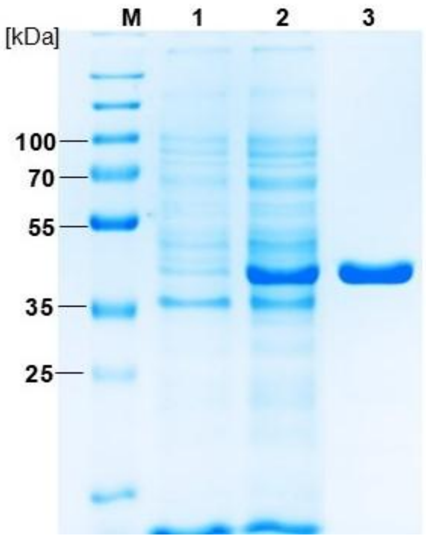

Figure S6. SDS-page analysis of purification of Epux2. Lane **M**: standard protein marker, lane **1**: *E. coli* BL21/pET24a cell extract, lane **2**: BL21/pET24a-Epux2 cell extract, lane **3**: Affinity chromatography purified Epux2.

Tabla S2. Averages and standard deviations of FT-IR spectroscopy transmittance values (%) of signals associated with polyurethane degradation. Impranil suspensions treated with Epux1, Epux2 and negative controls. Averages were obtained from three replicates with the Spectragryph software.

	**No enzymatic treatment**		**Epux1**		**Denatured Epux1**		**Epux2**		**Denatured Epux2**	
**Wavenumber (cm^{-1})**	**Mean**	**SD**	**Mean**	**SD**	**Mean**	**SD**	**Mean**	**SD**	**Mean**	**SD**
**1730**	84.71	1.41	92.08	0.17	85.72	3.30	90.88	1.68	87.35	2.48
**1560**	46.65	3.25	41.38	3.04	46.00	6.12	39.51	1.11	50.34	6.64
**1260**	91.90	1.00	97.71	0.42	93.36	2.48	97.13	1.84	94.32	2.09
**1040**	71.26	4.35	73.47	5.43	70.52	3.910	75.00	2.29	67.09	4.07
