# Supplementary material for: Enzymatic characterization and polyurethane biodegradation assay of two novel esterases isolated from a polluted river
Source: PLoS One. 2025 Jul 23;20(7):e0327637. doi: 10.1371/journal.pone.0327637 (PMC12286390; doi:10.1371/journal.pone.0327637)

Original SDS page gels.

Method used for capture: Gel Doc EZ Imager (BIO-RAD) documentation system, using a white screen.

1. Gel from **Fig S5.** Semi-native SDS–PAGE (12 % acrylamide). Only Epux1 (10 μg) and the lane marker (M) were used for **Fig S5.**

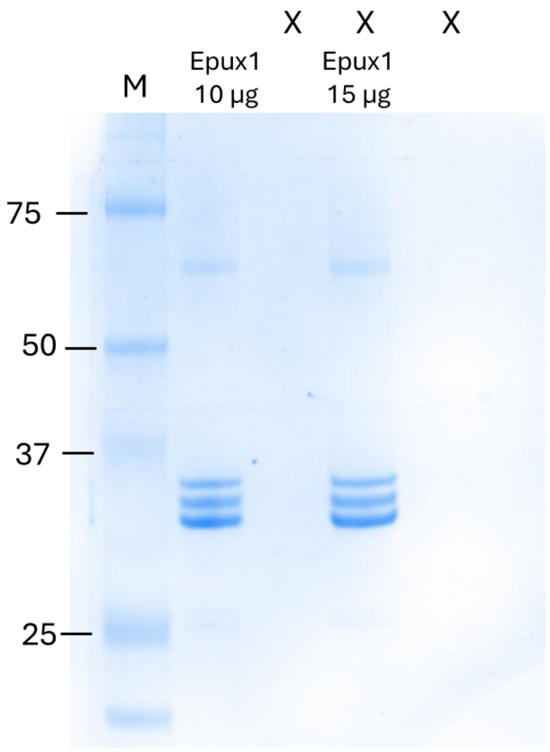

2. Gel of Epux2 purification (12 % acrylamide). Only lanes 1,2, and 4 were used for **Fig S6.** Lane **M:** standard protein marker, lane **1**: E. coli BL21/pET24a cell extract, lane **2**: BL21/pET24a-Epux2 cell extract, lane **3**: wash step, lane **4**: Affinity chromatography purified Epux2 after dialysis lanes **5-7**: fractions eluted from column chromatography.

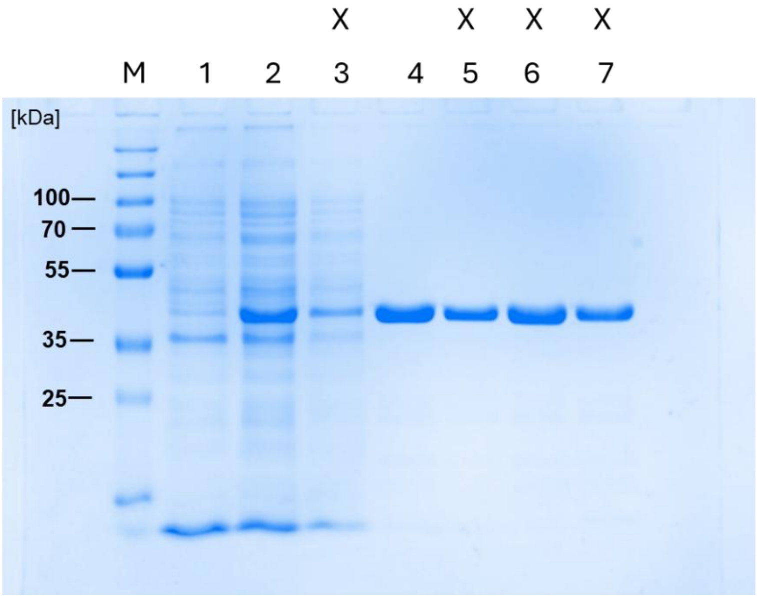

Supplement: S1 Raw images — (PDF) [file pone.0327637.s002.pdf]
