# Supplementary figures and images for: Enzymatic characterization and polyurethane biodegradation assay of two novel esterases isolated from a polluted river
Source: PLoS One. 2025 Jul 23;20(7):e0327637. doi: 10.1371/journal.pone.0327637 (PMC12286390; doi:10.1371/journal.pone.0327637)

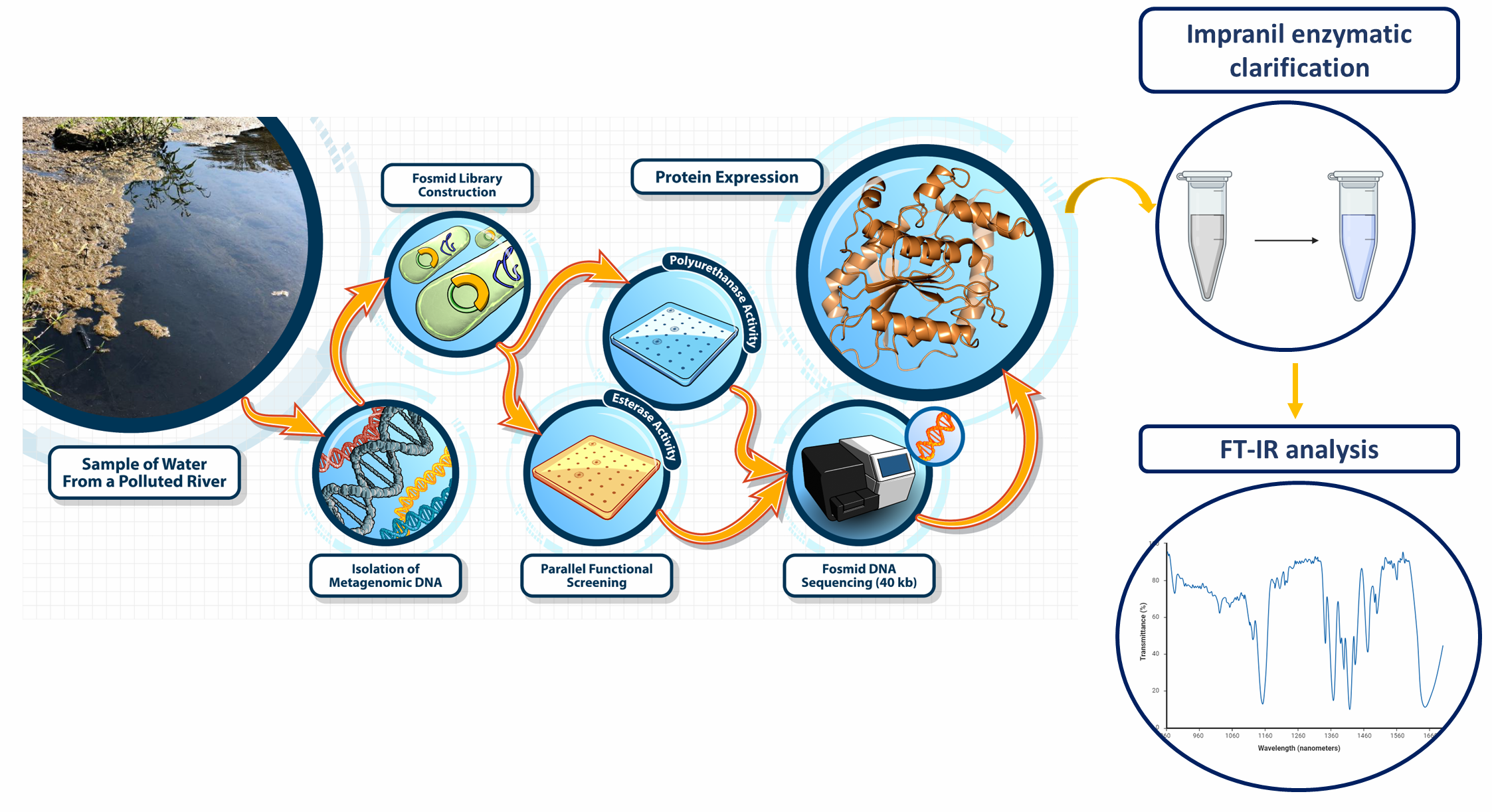

Supplement: S1 Fig — (TIF) [file pone.0327637.s003.tif]
